# The Parent Version of the Sensitivity to Pain Traumatization Scale (SPTS-P): A Preliminary Validation

**DOI:** 10.3390/children8070537

**Published:** 2021-06-24

**Authors:** Jaimie K. Beveridge, Maria Pavlova, Joel Katz, Melanie Noel

**Affiliations:** 1 Department of Psychology, University of Calgary, Calgary, AB T2N 1N4, Canada; mpavlova@ucalgary.ca (M.P.); melanie.noel@ucalgary.ca (M.N.); 2 Department of Psychology, York University, Toronto, ON M3J 1P3, Canada; jkatz@yorku.ca; 3 Alberta Children’s Hospital Research Institute, Calgary, AB T2N 4N1, Canada; 4 Hotchkiss Brain Institute, Calgary, AB T2N 4N1, Canada; 5 Mathison Centre for Mental Health Research and Education, Calgary, AB T2N 4Z6, Canada

**Keywords:** chronic pain, youth, trauma, parents, scale development, factor analysis

## Abstract

Sensitivity to pain traumatization (SPT) is defined as the propensity to develop responses to pain that resemble a traumatic stress reaction. To date, SPT has been assessed in adults with a self-report measure (Sensitivity to Pain Traumatization Scale (SPTS-12)). SPT may also be relevant in the context of parenting a child with chronic pain, as many of these parents report clinically elevated posttraumatic stress symptoms (PTSS). This study aimed to develop and validate a measure of parent SPT by adapting the SPTS-12 and evaluating its psychometric properties in a sample of parents whose children have chronic pain. In total, 170 parents (90.6% female) and children (aged 10–18 years, 71.2% female) were recruited from a tertiary chronic pain program. Parents completed the parent version of the SPTS-12 (SPTS-P) and measures of PTSS, depression, anxiety and anxiety-related constructs, and parenting behaviors. Youth completed measures of pain. Consistent with the SPTS-12, the SPTS-P demonstrated a one-factor structure that accounted for 45% of the variance, adequate to good reliability and moderate construct validity. Parent SPT was positively related to their protective and monitoring behaviors but was unrelated to youth pain intensity, unpleasantness, and interference. These results provide preliminary evidence for the psychometric properties of the SPTS-P and highlight the interaction between parent distress about child pain and parent responses to child pain.

## 1. Introduction

Posttraumatic stress disorder (PTSD) is a mental health condition that develops after exposure to a traumatic event or major life stressor (e.g., illness). Symptom clusters include re-experiencing, avoidance, negative alterations in cognitions and mood, and hyperarousal, all of which can cause significant distress and impairment [1]. Some individuals experience symptoms of a traumatic stress reaction that do not meet diagnostic criteria. Nevertheless, even these subsyndromal PTSD symptoms (PTSS) can have a deleterious impact on functioning. Both PTSD and PTSS have been shown to co-occur at high rates with chronic pain in youth and adults [2,3,4]. Current conceptual models for this co-occurrence suggest that symptoms of chronic pain and PTSD may interact to maintain one another (mutual maintenance model) and that certain psychological factors may put individuals at-risk of developing both conditions when exposed to painful or traumatic life events (shared vulnerability model) [3,5].

Sensitivity to pain traumatization (SPT) is a construct proposed to be a vulnerability factor for co-occurring chronic pain and PTSS. It was developed by evaluating the underlying factor structure of three pain-related anxiety constructs (i.e., anxiety sensitivity, pain anxiety, pain catastrophizing) in a sample of adults undergoing major surgery [6]. Among these constructs, one higher-order factor was identified that described features of a traumatic stress reaction, including experiencing and re-experiencing pain, avoidance of pain, and hyperarousal to pain, and was related to PTSS before surgery and to chronic pain 12-months post-surgery. The authors conceptualized this higher-order factor as SPT and defined it as a predisposition to developing anxiety-related somatic, cognitive, emotional, and behavioral responses to pain that resemble the features of a traumatic stress reaction [6]. Importantly, SPT scores, but not scores on the measure of PTSS, were found to predict the development of chronic postsurgical pain. Thus, the authors clarify that SPT is distinct from PTSD but may, in part, explain the high rates of PTSS among individuals with chronic pain. Specifically, SPT represents a vulnerability to develop traumatic-stress-like reactions to pain. Based on these findings, Katz et al. [7] developed a self-report measure of SPT (SPTS-12) using item response theory. The SPTS-12 was validated in samples of undergraduate students, including students with ongoing pain, and a sample of adult patients who had undergone surgery. It was found to have a one-factor structure, excellent reliability, and moderate to excellent validity in the community and clinical samples and in individuals who were pain-free or reporting ongoing pain [7].

A measure of SPT may also be relevant for parents, particularly those whose children have chronic pain. To date, research on the mental health of parents whose children have chronic pain has primarily focused on symptoms of depression and anxiety [8]. This research has shown that elevated depressive and anxiety symptoms are prevalent among parents [9,10], integrally related to the child’s pain-related functioning [8], and thus important to assess in research and clinical practice. In fact, at least one self-report measure (i.e., the Bath Adolescent Pain—Parental Impact Questionnaire [11]) has been developed to assess the impact of parenting a youth with chronic pain and includes subscales that measure depressive and anxiety symptoms related to living with a child in pain. Moreover, psychological treatments are now being developed that target distress, specifically depressive symptoms, among parents of youth with chronic pain [12].

Emerging research is demonstrating that PTSS is also prevalent among parents of youth with chronic pain. Specifically, Noel et al. [2] have shown that parents of youth with chronic pain report significantly higher PTSS, and are also more likely to meet clinical cut-offs for PTSD than parents of youth without chronic pain. Importantly, higher PTSS in parents is related to greater pain intensity and interference in youth with chronic pain [2,13]. These results are consistent with other pediatric health conditions (e.g., diabetes, cancer), wherein rates of PTSD and PTSS are prevalent among parents and related to their child’s functioning [14]. While the high rates of PTSS among parents of youth with chronic pain may reflect a more general vulnerability for mental health problems in these families, findings also suggest that, for some parents, the very experience of parenting a child with a health condition, such as chronic pain, is a traumatic event and may evoke a traumatic-stress-like reaction. In fact, when asked about the worst traumatic event experienced in their life, 9% of parents in Noel et al.’s study reported their child’s chronic pain to be their worst event [2]. These parents may perceive their child’s pain to be threatening and thus respond with protective and monitoring behaviors. Indeed, research has shown that parents with higher distress about their child’s pain are more likely to be protective (e.g., restrict their child’s activities) [15,16], and this is related to greater pain and impairment among youth with chronic pain [17,18,19]. These parents may be at risk of developing PTSS related to their child’s pain. In this way, parent SPT may in part explain the high rates of PTSS among parents of youth with chronic pain. However, parents may also develop responses to their child’s pain that resemble a traumatic stress reaction without developing PTSD, similar to parents who experience depressive and/or anxiety symptoms related to their child’s pain but do not generalize these symptoms outside of this parenting experience to meet diagnostic criteria for a depressive or anxiety disorder.

Given that trauma-related symptomatology and its treatment are distinct from depression and anxiety [1], similar measures and treatments that have been developed for depressive and anxiety symptoms in parents of youth with chronic pain are also needed for PTSS. SPT is a novel construct that assesses the features of a traumatic stress reaction in the context of pain. The aims of the current study were to develop a measure of parent SPT by adapting the SPTS-12 and evaluating its psychometric properties in a clinical sample of youth with chronic pain and their parents. We hypothesized that the parent version of the Sensitivity to Pain Traumatization Scale (SPTS-P) would have a one-factor structure similar to the SPTS-12 and demonstrate excellent reliability and construct validity. Specifically, we hypothesized that the SPTS-P would have excellent internal consistency and test-retest reliability and that parent scores on the SPTS-P would be more related to their scores on a measure of PTSS (convergent validity) than their scores on a measure of depressive symptoms (discriminant validity) and would also be related to their protective and monitoring behaviors as well as their child’s pain-related outcomes.

## 2. Materials and Methods

### 2.1. Participants

Youth and parents were recruited from the headache, complex pain, and abdominal pain clinics of a tertiary chronic pain program at a pediatric hospital in Western Canada. Families were recruited as part of the Pain and Mental Health in Youth (PATH) study, a broader program of research examining a myriad of cognitive, behavioral, neurobiological, and social factors in the co-occurrence of chronic pain and internalizing mental health disorders in youth. To date, studies have used data from the PATH study to examine diagnostic uncertainty, sleep disturbances, daily relations between parent and child functioning, intolerance of uncertainty, attentional biases, and adverse childhood experiences [20,21,22,23,24,25,26]. As such, the aims of the current study are distinct from these published studies.

Youth were eligible to participate if they were between 10 and 18 years of age, had chronic pain (>3 months, in accordance with the current definition of chronic pain) [27] that was ongoing at the time of recruitment, had English language fluency, and were able to access the internet. Exclusion criteria for youth included the known presence of a serious physical or mental health condition (e.g., cancer, arthritis, psychotic disorder, cognitive impairment, developmental disorder). Parents were eligible to participate if they had English language fluency, were able to access the internet and were the legal guardian of the youth. Thus, the term ‘parent’ in this study refers to any adult caregiver who has assumed a primary parenting role of the child.

Recruitment took place between January 2017 and March 2020. In total, 360 families were contacted and invited to participate in the study. Of these, 63 were not eligible, and 107 either did not want to participate or could not be reached by phone or email again, after the initial contact, to be enrolled in the study. Of the 190 parent-child dyads who consented, 8 withdrew from the study before completing the baseline survey, 1 was not able to participate due to the COVID-19 pandemic, 2 parents were enrolled twice (with a different child), and 2 parents did not complete the baseline survey. For the current study, data from families in which the parent failed to respond to all items of the SPTS-P at baseline (*n* = 7) were also excluded. Thus, the final sample included 170 parents and youth. For the test-retest reliability analysis (described below), data from families in which the parent failed to respond to all items of the SPTS-P at follow-up (*n* = 3) were excluded. In addition, from the baseline sample, 2 families withdrew from the study before follow-up, 4 were lost to follow-up, and 11 did not complete the follow-up survey. Thus, the final sample for the test-retest reliability analysis included 150 parents.

### 2.2. Procedure

All study procedures were reviewed and approved by the university’s health research ethics board. Clinic staff identified potential participants and obtained consent to be contacted for research purposes from parents at the time of booking their child’s initial pediatric chronic pain clinic appointment. The study team then contacted potential participants via phone or email with information about the study. Interested parent-child dyads were screened for eligibility over the phone, and a verbal informed consent procedure was conducted with the dyad where the purpose of the study was outlined, and their agreement to participate was obtained. In addition, written informed consent was obtained from parents and youth through online consent forms that were emailed after the verbal consent procedure.

Once enrolled, parents and youth each completed an online survey consisting of self-report measures. Surveys were administered and completed using Research Electronic Data Capture (REDCap), a secure web-based data collection site [28]. Follow-up for the PATH study took place approximately 3 months after baseline (*M* = 107.5 days, *SD* = 17.2 days), as this is the typical follow-up timepoint for pediatric chronic pain treatment programs [29,30]. At this timepoint, parents and youth completed an online survey that contained the same self-report measures as the baseline survey. Only parent follow-up surveys were used in the current study to assess the test-retest reliability of the SPTS-P. Parents and youth each received an honorarium ($10 or $20 CAD gift card) for each survey completed.

### 2.3. Measures

#### 2.3.1. Sociodemographic Form

Parents provided sociodemographic information on their age, gender, race/ethnicity, relationship to the child, marital status, annual household income, and education as well as their child’s age, gender, and race/ethnicity for descriptive purposes.

#### 2.3.2. Sensitivity to Pain Traumatization Scale-Parent Version (SPTS-P)

**Figure 1:**
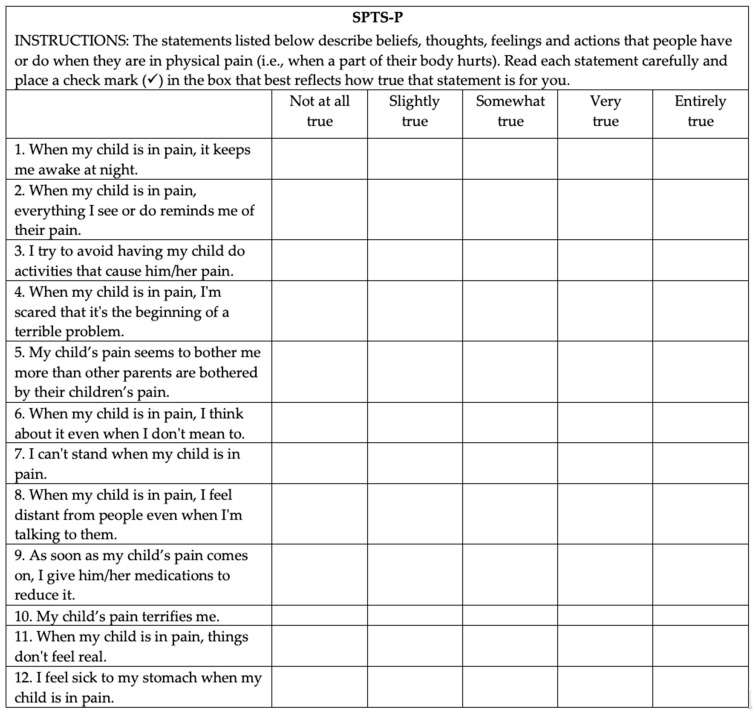
Final version of the Sensitivity to Pain Traumatization Scale-Parent Version (SPTS-P). Each of the 12 items are rated on a 5-point (0–4) scale where 0 = not at all true; 1 = slightly true; 2 = somewhat true; 3 = very true; 4 = entirely true.

The original SPT measure, the SPTS-12 [7], was developed using item response theory, classical test theory, and exploratory factor analysis. A list of items was compiled from valid and reliable measures that assess vari-ous aspects of pain, anxiety, and trauma as well as a review of the literature. Items were selected for inclusion in the final scale on the basis of item characteristic curves, option characteristic curves, and their representation of the theoretical dimensions of SPT. For the current study, the SPTS-12 was adjusted to be consistent with the parental role and sensitivity of parents to develop responses to their child’s pain that resemble a traumatic stress reaction by rephrasing the items. For example, the stems of relevant items were changed from ‘When I am in pain…’ to ‘When my child is in pain…’. The original content/meaning of all items was retained from the original measure. This method of adapting a parent version of a measure is consistent with previous studies that have adapted pain-related measures for use in parent populations, such as the widely-used Pain Catastrophizing Scale-Parent Version [31]. The final version of the SPTS-P is shown in Figure 1. The SPTS-P consists of 12 statements that describe beliefs, thoughts, feelings, and actions that parents can have when their child is in pain. Parents were asked to rate the extent to which each statement was true for them on a 5-point Likert-type scale (0 = ‘not at all true’ to 4 = ‘entirely true’). The ratings for each item were summed and yield a total score that can range from 0 to 48. Higher scores indicated a greater propensity for parents to respond to their child’s pain with anxiety-related somatic, cognitive, emotional, and behavioral responses that are similar to a traumatic stress reaction. The original SPTS-12 has demonstrated good to excellent reliability (*α*s = 0.84–0.90), excellent concurrent validity, and moderate construct validity among adults with and without chronic pain, in community and clinical (i.e., patients undergoing major surgery) samples [7]. The psychometric properties for the SPTS-P are provided in the results section of the current article.

#### 2.3.3. PTSD Checklist for DSM-5 (PCL-5) with Criterion A

The PCL-5 with Criterion A assessed parent exposure to traumatic events and their current PTSS [32]. This measure was administered to examine the convergent validity of the SPTS-P, by correlating scores on the SPTS-P with scores on a measure of a construct (i.e., PTSS) that is theoretically related, given the overlapping symptoms of a traumatic-stress reaction, but not specific to pain. This measure first asks respondents to briefly describe the worst traumatic event they have experienced that continues to bother them in a textbox. Items then assess the extent to which this event meets Criterion A (i.e., exposure to actual or threatened death, serious injury, or sexual violence [1]). These items were not considered as part of the current study, as we were interested in the presence of symptoms related to PTSD and not the diagnosis of PTSD. As such, and consistent with previous research that has used this measure to assess PTSS in parents of youth with chronic pain [2,13,33], participants did not have to meet Criterion A to have their scores on the PCL-5 included in the analyses. Respondents were asked to keep this event in mind as they rated how much 20 symptoms, which were specific to DSM-5 diagnostic criteria for PTSD [1], have bothered them in the past month on a 5-point Likert-type scale (0 = ‘not at all’ to 4 = ‘extremely’). Total scores for the PCL-5 were obtained by summing the ratings for the 20 items (range: 0–80). Higher scores reflected greater parent PTSS, with a score of 33 suggested by the National Center for PTSD as the clinical cut-off [34]. Similar to previous research [2,13,33], we used the clinical cut-off for descriptive purposes to identify the percentage of parents reporting clinically-elevated PTSS, and the continuous total score for the main analyses. This measure was developed at the National Center for PTSD and is one of the most widely-used self-report measures of PTSS in research and clinical settings [35]. Moreover, the PCL has been extensively validated since its first iteration, and the most recent version, the PCL-5, has excellent internal consistency, good test-retest reliability, and has demonstrated construct validity in a civilian population [36]. It has also been used in previous research with parents of youth with chronic pain [2,33] and showed excellent internal consistency in this study (*α* = 0.93).

#### 2.3.4. Hospital Anxiety and Depression Scale (HADS)

The HADS assessed parent symptoms of depression (HADS-D) and anxiety (HADS-A) [37]. The HADS-D was administered to examine the discriminant validity of the SPTS-P, by correlating scores on the SPTS-P with scores on a measure of a construct (i.e., depression) that may be related to SPT, due to the general levels of distress that both may capture, but is theoretically distinct, given the differences in depressive vs. traumatic-stress-like reactions. Consistent with the development study of the SPTS-12 [7], the HADS-A was administered to examine the relation of the SPTS-P with a measure of general anxiety. The HADS consists of 14 items that ask about anxiety and depressive symptoms experienced in the past week. Items were rated on a 4-point Likert-type scale, with each item having different anchors (range: 0–3). Total scores for anxiety and depressive symptoms were obtained by summing the ratings of the relevant items for each subscale (range: 0–21). Higher scores indicated greater symptoms of parent anxiety or depression. Given that many parents of youth with chronic pain report their own chronic pain condition [33,38], the HADS was chosen for this study because it was developed to assess anxiety and depression among individuals with physical health concerns by excluding symptoms that may confound diagnosis due to their overlap with these conditions. The HADS-A and HADS-D have demonstrated good reliability and good to very good concurrent validity in patient (e.g., primary care, cancer) and community populations [39]. In this study, both the HADS-A and HADS-D showed good internal consistency (both *α*s = 0.82).

#### 2.3.5. Anxiety Sensitivity Index-3 (ASI-3)

The ASI-3 assessed the physical, cognitive, and social concerns of anxiety sensitivity (i.e., the fear of anxious arousal sensations and their consequences) among parents [40]. This measure was administered to examine the relation of the SPTS-P with measures of pain-related anxiety constructs similar to the ones used in the original development studies of SPT and the SPTS-12 [6,7]. The ASI-3 asked respondents to rate how much they agree with 18 statements on a 5-point Likert-type scale (0 = ‘very little’ to 4 = ‘very much’). Total scores were obtained by summing the ratings for each item (range: 0–72), with higher scores indicating greater anxiety sensitivity in parents related to their own sensations of anxious arousal. The ASI-3 has excellent reliability and demonstrated construct validity among patients with anxiety disorders and non-clinical populations [40,41,42]. The ASI-3 demonstrated excellent internal consistency in the current study (*α* = 0.90).

#### 2.3.6. Pain Anxiety Symptoms Scale-20 (PASS-20)

The PASS-20 assessed parents’ pain-related anxiety symptoms [43]. This measure was administered to examine the relation of the SPTS-P with measures of pain-related anxiety constructs similar to the ones used in the original development studies of SPT and the SPTS-12 [6,7]. The PASS-20 is comprised of 20 items that reflect different responses to pain. Respondents were asked to rate how often they engage in each thought or activity on a 6-point Likert-type scale (0 = ‘never’ to 5 = ‘always’). Total scores were obtained by summing the ratings for each item (range: 0–100), with higher scores indicating higher levels of pain-related anxiety in parents related to their own pain. The PASS-20 has excellent reliability and demonstrated construct validity among patients with chronic pain and non-clinical samples [43,44]. The PASS-20 showed excellent internal consistency in the current study (*α* = 0.92).

#### 2.3.7. Pain Catastrophizing Scale for Parents (PCS-P)

The PCS-P measured the parent’s catastrophic thinking about their child’s pain (i.e., ruminating about the child’s pain, magnifying the threat of the child’s pain, and focusing on feelings of helplessness when the child is in pain) [31]. This measure was administered to examine the relation of the SPTS-P with measures of pain-related anxiety constructs similar to the ones used in the original development studies of SPT and the SPTS-12 [6,7]. The PSC-P asked parents to rate the extent to which they experience 13 different thoughts and feelings when their child is in pain on a 5-point Likert-type scale (0 = ‘not at all’ to 4 = ‘extremely’). Total scores were obtained by summing the ratings for each item (range: 0–52), with higher scores indicating greater parent catastrophizing about child pain. The PCS-P has excellent reliability and demonstrated construct validity among parents of school children and parents of youth with chronic pain [31]. The PCS-P showed excellent internal consistency in the current study (*α* = 0.91).

#### 2.3.8. Adult Responses to Children’s Symptoms (ARCS)

The Protect and Monitor subscales of the ARCS with a pain-specific stem (i.e., “When your child has pain…”) measured the protective and monitoring behaviors of parents [45]. This measure was administered to examine the construct validity of the SPTS-P, by correlating scores on the SPTS-P with scores on a measure of parenting behaviors that we hypothesized would be significantly related to parent SPT. This definition of construct validity is consistent with the COSMIN (COnsensus-based Standards for the selection of health Measurement INstruments) Taxonomy of Measurement Properties [46]. On this measure, parents were asked to rate how often they engage in protective and monitoring behaviors when their child is in pain on a 5-point Likert-type scale (0 = ‘never’ to 4 = ‘always’). The developmentally-sensitive scoring system put forth by Noel et al. [47] for a combined sample of children and adolescents (ages 7–18) was used to calculate scores for the Protect and Monitor subscales. Specifically, scores for the Protect subscale (13 items) and Monitor subscale (4 items) were computed as averages, with higher scores indicating greater incidence of the behavior. The Protect and Monitor subscales were exclusively chosen for the current study because they demonstrate good reliability (*α* = 0.87 and 0.79, respectively) as well as sensitivity and responsiveness to change following psychological treatment for pediatric chronic pain [47,48]. The Protect and Monitor subscales also have evidenced factor validity for the combined sample of children and adolescents [47]. The remaining subscales of the ARCS (Minimize and Distract) have questionable reliability (*α* = 0.63 and 0.70, respectively) and were not sensitive or responsive to change [47,48]. Thus, these subscales were not included in the current study. The Protect subscale showed excellent internal consistency (*α* = 0.90), and the Monitor subscale showed good internal consistency (*α* = 0.85) in this study.

#### 2.3.9. Pain Questionnaire

Items from the Pain Questionnaire assessed the following youth pain characteristics: location(s), frequency, duration, intensity, and unpleasantness [49]. The pain location item asked respondents to select the location(s) of their pain from a checklist of 6 options (e.g., stomach, head, other). The pain frequency item asked the respondent to rate how often they had pain in the past week on a 5-point scale (0 = ‘not at all’ to 4 = ‘daily’). The daily pain duration item assessed how long the respondent’s pain usually lasts on a 4-point scale (0 = ‘less than 1 hour’ to 3 = ‘all day’). The total duration of the youth’s chronic pain was assessed with an item that asked respondents to indicate how long they have had their pain in years and months. These items were used for descriptive purposes in the current study to characterize the pain experiences of the youth. Pain intensity was measured with a well-validated and reliable 11-point Numerical Rating Scale (0 = ‘no pain’ to 10 = ‘worst pain possible’) [50]. Pain unpleasantness was measured with a well-validated item that asked the respondent to rate the extent to which pain bothers or upsets them on a 5-point Likert-type scale (1 = ‘not at all’ to 5 = ‘very much’) [51]. These items, along with the measure of pain interference, were administered to examine the construct validity of the SPTS-P, by correlating parent scores on the SPTS-P with youth scores on measures related to their chronic pain, which we hypothesized would be significantly related to parent SPT.

#### 2.3.10. Pain Interference Short-Form of the Patient-Reported Outcomes Measurement Information System (PROMIS) Pediatric Profile-25

This 4-item short-form asked the respondent to rate the extent to which pain interfered with various activities in the past week on a 5-point Likert-type scale (1 = ‘never’ to 5 = ‘almost always’). Total scores were obtained by transforming the summed ratings for each item into standardized *T*-scores. Higher scores indicated greater youth pain interference. This form was developed by the National Institutes of Health using item response theory and has been validated in youth with chronic pain [52]. It demonstrated good internal consistency in this study (*α* = 0.82).

### 2.4. Statistical Analyses

Analyses were conducted in RStudio (Version 1.2.5019; Boston, MA, USA) and IBM SPSS (Version 24; Armonk, NY, USA). Exploratory factor analysis using the polychoric correlation matrix and principal axis factoring estimation was conducted in RStudio using the “psych,” “Hmisc,” and “poly_cor” packages to evaluate the scale items and factor structure of the SPTS-P. The number of factors to retain was determined through examination of the scree plot, parallel analysis, Velicer’s minimum average partial (MAP) test, the root mean square residual (RMR), and the ratio of the first initial eigenvalue to the second initial eigenvalue. Although the SPTS-P was based on an existing measure, exploratory factor analysis was chosen over confirmatory factor analysis because the SPTS-P is a new measure, distinct from the SPTS-12, that assesses a unique construct (i.e., *parent* SPT) in a novel population (i.e., parents of children with chronic pain).

The types of traumatic events reported by parents were independently coded by 2 coders according to categories used by Noel et al. [2]. The remainder of the analyses were conducted in SPSS. Descriptive statistics were conducted to characterize the sample. Cronbach’s alpha (*α*), corrected item-total correlations, and Cronbach’s *α*-if-item-deleted correlations were conducted to examine the internal consistency of the SPTS-P. Spearman correlations were conducted for the remaining analyses. Specifically, correlations between baseline and follow-up SPTS-P scores evaluated the test-retest reliability of the SPTS-P, correlations between SPTS-P scores and scores on the HADS-A, ASI-3, PASS-20, and PCS-P evaluated the relation of parent SPT with relevant measures of general and pain-related anxiety constructs, correlations between SPTS-P scores and PCL-5 scores evaluated the convergent validity of the SPTS-P, correlations between SPTS-P scores and HADS-D scores evaluated the discriminant validity of the SPTS-P, and correlations between scores on the SPTS-P and parent scores on the ARCS subscales and youth scores on the pain measures further evaluated the construct validity of the SPTS-P. For the convergent and discriminant validity analyses, a correlation of *r* ≥ 0.30 between the SPTS-P and PCL-5 was considered to demonstrate adequate convergent validity while a correlation of *r* ≤0.30 between the SPTS-P and HADS-D was considered to demonstrate adequate discriminant validity. These interpretations were based on recently published considerations and meta-analytic findings [53,54,55] that the effect size interpretations originally proposed by Cohen [56,57] are not accurate for psychological research, and that effect sizes of *r* = 0.10, 0.20, and 0.30 can be interpreted, respectively, as relatively small, typical, and relatively large. The magnitude of the difference between the correlation coefficients for the SPTS-P and PCL-5 and for the SPTS-P and HADS-D was also evaluated [58], with a significantly larger correlation between the SPTS-P and PCL-5 than the SPTS-P and HADS-D indicating good convergent and discriminant validity.

As noted, participants who did not complete the baseline and/or follow-up surveys were excluded from the current study. Missing data that resulted from a respondent failing to respond to one or more items of a study measure was handled using multiple imputation. Specifically, the Multiple Imputation procedure through the Missing Value Analysis module in SPSS, which uses a linear regression model, was run with 20 imputations as recommended by Enders [59] and Graham, Olchowski, and Gilreath [60]. Overall, missing data ranged from a low of 0.6% on the HADS-A and ARCS Monitor to a high of 5.9% on the PCS-P. Missing data were determined by Little’s MCAR test [61] to be missing completely at random, χ^2^ (120) = 120.48, *p* = 0.470.

## 3. Results

### 3.1. Sample Characteristics

The sociodemographic and pain characteristics of the sample are reported in Table 1. The majority of parents were female (90.6%), White (85.3%), married or common-law (78.8%), and identified as the biological parent of the child (97.6%). Parents ranged in age from 30 to 64 years, with an average age of 45.03 years (*SD* = 5.62 years). Most parents had at least a college or Bachelor’s degree (64.7%) and an annual household income greater than $90,000 CAD (57.6%). The majority of youth were also female (71.2%) and White (80.0%). Youth ranged in age from 10 to 18 years, with an average age of 14.32 years (*SD* = 2.22 years), and reported an average pain duration of 3.23 years (*SD* = 3.08 years; range = 3 months to 17 years). Almost half (47.6%) of youth reported having pain on a daily basis, and the majority (56.5%) reported that their pain lasts for half of the day or the whole day. The most highly endorsed pain locations among youth were head (72.9%), muscles and joints (25.3%), and other (24.1%). In total, 6 parents (3.5%) met the clinical cut-off for PTSD. The types of traumatic events reported by parents are reported in Table 2.

### 3.2. Factor Analysis

Table 3 summarizes the means (*M*) and standard deviations (*SD*) of the 12 items of the SPTS-P as well as Cronbach’s *α*-if-item-deleted, corrected item-total correlations, factor loadings, and communalities for the one-factor solution.

Bartlett’s test of sphericity was significant (*p* < 0.001), indicating that the factor analysis was appropriate. The Kaiser–Meyer–Olkin (KMO) measure of sampling adequacy indicated that the factor analysis was good (KMO = 0.89). All items loaded onto one factor at a value of 0.32 or greater [62]. An examination of the scree plot suggested that a one-factor solution was appropriate. Results from a parallel analysis using real-data eigenvalues and Velicer’s MAP test also yielded a one-factor solution. The RMR was 0.065, indicating a good fit and providing support for the one-factor model. In addition, the ratio of the first (5.39) to the second (0.49) eigenvalue was >4. The one-factor model accounted for 44.9% of the variance.

### 3.3. Reliability

#### 3.3.1. Internal Consistency

Internal consistency of the SPTS-P was *α* = 0.86. Cronbach’s *α*-if-item-deleted analyses indicated the reliability of the SPTS-P would be slightly improved if item 9 was deleted (*α* = 0.87). Deletion of any other item would not improve the internal consistency of the scale (*α* = 0.84–0.86). Corrected item-total correlations ranged from 0.307 to 0.688.

#### 3.3.2. Test-Retest Reliability

SPTS-P scores at baseline were significantly correlated with SPTS-P scores at follow-up, *r*(150) = 0.727, *p* < 0.001.

### 3.4. Validity

Table 4 and Table 5 report correlation matrices for the SPTS-P and related constructs.

#### 3.4.1. Convergent Validity

The SPTS-P was significantly correlated with the PCL-5, *r*(170) = 0.310, *p* < 0.001. The coefficient of determination (*r*^2^) showed that 9.61% of the variance was shared between the SPTS-P and the PCL-5.

#### 3.4.2. Discriminant Validity

The SPTS-P was significantly correlated with the HADS-D, *r*(170) = 0.199, *p* = 0.009. The coefficient of determination (*r*^2^) showed that 3.96% of the variance was shared between the SPTS-P and the HADS-D. The magnitude of the correlation between the SPTS-P and PCL-5 was not significantly greater than the correlation between the SPTS-P and HADS-D (*z* = 1.72, *p* = 0.086).

#### 3.4.3. Construct Validity with Anxiety-Related Constructs

The SPTS-P was significantly correlated with the ASI-3, *r*(170) = 0.298, *p* < 0.001, the PASS-20, *r*(170) = 0.393, *p* < 0.001, the PCS-P, *r*(170) = 0.742, *p* < 0.001, and the HADS-A, r(170) = 0.268, p < 0.001.

#### 3.4.4. Construct Validity with Parenting Behaviors

The SPTS-P was significantly correlated with the Protect, *r*(170) = 0.608, *p* < 0.001, and Monitor, *r*(170) = 0.458, *p* < 0.001, subscales of the ARCS.

#### 3.4.5. Construct Validity with Youth Pain

The SPTS-P was not significantly related to youth ratings of their pain intensity, *r*(170) = −0.024, *p* = 0.763, pain unpleasantness, *r*(170) = 0.055 *p* = 0.479, or pain interference, *r*(170) = 0.068, *p* = 0.382.

### 3.5. Correlation with Sociodemographic Variables

Parent SPT scores were not significantly related to parent age, *r*(165) = −0.015, *p* = 0.848, parent education, *r*(170) = −0.039, *p* = 0.613, annual household income, *r*(148) = −0.066, *p* = 0.428, or youth age, *r*(170) = −0.036, *p* = 0.643. Parent SPT scores did not differ significantly between parents of girls and parents of boys, *t*(165) = −1.62, *p* = 0.108.

## 4. Discussion

This study developed and examined the psychometric properties of a measure of parent sensitivity to pain traumatization, which we defined as the propensity of parents to develop anxiety-related responses to their child’s pain that resemble features of a traumatic stress reaction. Similar to the original adult measure, the SPTS-P was found to have a one-factor structure, adequate to good reliability, and moderate construct validity. Specifically, parent scores on the SPTS-P were related to their scores on measures of PTSS, depressive symptoms, and parenting behaviors (i.e., more protective and monitoring responses). Notably, correlations between these measures suggest that parent SPT is a unique construct that is related to, but distinct from, parent PTSS, parent depressive symptoms, and parent behavioral responses to child pain. These results provide preliminary evidence for the construct of parent SPT as well as the psychometric properties of the SPTS-P as a measure of parent SPT. Contrary to hypotheses, parent SPT was not related to youth chronic pain outcomes.

Exploratory factor analysis demonstrated that the 12 items of the SPTS-P loaded onto a single factor that accounted for 45% of the variance and had good fit indices. This is consistent with the original SPTS-12, which demonstrated a one-factor solution in clinical and non-clinical samples that accounted for 46.1% to 54.7% of the variance. In the current study, item 9 had the lowest loading onto the factor. This is consistent with the original development paper, in which item 9 provided the least discrimination in the item response theory analyses and had the lowest loadings onto the one-factor solution. Item 9 describes a specific behavior (‘As soon as my child’s pain comes on, I give him/her medications to reduce it’) and is thus distinct from the other items, which describe internal thoughts, feelings, and experiences resembling the features of a traumatic stress response. Item 9 was included in the original scale to measure pain avoidance and was retained by the authors, despite its poor fit, because there was not a suitable replacement that would maintain the face validity of the scale and adequately capture the pain avoidance dimension of SPT. Due to the preliminary nature of the current study and to maintain consistency with the original scale, we retained item 9 in the SPTS-P. Moreover, results indicated that the internal consistency of the scale would only marginally improve if item 9 was deleted (*α* would increase from 0.86 to 0.87). However, future research should continue to evaluate the fit of item 9 with the SPTS-P or consider additional avoidance items not related to medication use. Overall, the SPTS-P demonstrated good internal consistency and adequate test-retest reliability.

In regard to convergent and discriminant validity, a large correlation was found between the SPTS-P and the measure of PTSS (PCL-5), whereas a small-to-medium correlation was found between the SPTS-P and the measure of depressive symptoms (HADS-D). This is consistent with the SPTS-12 and demonstrates moderate convergent and discriminant validity, as the SPTS-P was more closely correlated with a construct that is theoretically related to SPT but not directly related to pain (i.e., PTSS) than a construct that is related to SPT but theoretically distinct (i.e., depressive symptoms). However, the correlation between the SPTS-P and the PCL-5 was not significantly greater than the correlation between the SPTS-P and the HADS-D, as would be expected for good convergent and discriminant validity. This suggests that the SPTS-P demonstrated moderate convergent and discriminant validity. Of note, the SPTS-P was also significantly correlated with the measure of anxiety symptoms (HADS-A). These significant relations among the SPTS-P and measures of depressive, anxiety, and posttraumatic stress symptoms likely reflect a level of general emotional distress that is shared among these internalizing mental health disorders and common among parents of youth with chronic pain [9,19,63].

Parents with higher scores on the SPTS-P reported engaging in more protective and monitoring responses when their child is in pain than parents with lower SPTS-P scores. Although they were not related to youth pain in the current study, extensive literature has shown that these behaviors tend to be related to worse pain and impairment among youth with chronic pain and are thus widely considered to be maladaptive [23,64,65,66,67,68]. Current psychological treatments for the management of pediatric chronic pain focus on reducing these parent behaviors, and these behaviors have been shown to be responsive to change in randomized controlled trials [69]. The results of the current study add to a growing body of literature demonstrating the critical importance of parent distress in pediatric chronic pain. Indeed, emerging research shows that parents with greater depressive symptoms, anxiety symptoms, and catastrophic thinking related to their child’s pain engage in more maladaptive parenting behaviors, which in turn predict worse child pain outcomes [18,19]. Moreover, these same parent distress variables have been shown to interfere with children’s response to psychological treatment for chronic pain, suggesting that parents with higher distress may have difficulty implementing treatment recommendations, including reducing their maladaptive behaviors [70]. Our results add to this research by demonstrating that parents with higher levels of traumatic distress are also more likely to engage in maladaptive parenting behaviors. Overall, findings to date suggest that parent distress should be a critical treatment target for pediatric chronic pain. Given these recent findings, Palermo et al. [12,71] evaluated the effectiveness of problem-solving skills training for parents of youth with chronic pain, which specifically aimed to reduce parent distress. The treatment led to improvements in mental health symptoms of both parents and children.

Despite the increasing attention to parent distress in the pediatric chronic pain literature, little attention has been given to parent trauma. The few studies that have examined parent trauma in the context of pediatric chronic pain have found that clinically-elevated PTSS are prevalent among these parents (e.g., in 20% vs. 1% of parents whose children do not have chronic pain) and play an important role in the child’s pain experience [2,13,33]. Of importance to parent SPT, 9% of parents in one study reported their child’s chronic pain as their worst traumatic life event [2]. A smaller proportion of parents (1.2%) in the current study reported their child’s chronic pain as their worst traumatic event. In addition, a smaller percentage of parents (3.5%) met the clinical cut-off for PTSD. These findings suggest that traumatic-stress-like symptoms, in general, and in response to the child’s chronic pain, may not have been as prevalent in the current sample. Further research in other samples of parents of youth with chronic pain is needed to understand better the prevalence and role of parent SPT in pediatric chronic pain. Qualitative research has begun to elucidate the traumatic aspects of parenting a child with chronic pain. In a study by Jordan et al. [72], one parent described her inability to take away her child’s chronic pain as “worse than frustration…. It’s like being tortured” (p. 52). Investigations of parent SPT provide an opportunity to expand on our understanding of parental traumatic stress in the context of pediatric chronic pain by examining this novel construct that is related to traumatic stress but unique to the pain experience. Of particular importance is whether the SPTS-P will be able to identify parents at risk of developing high levels of traumatic stress and/or clinically-elevated PTSS in response to their child’s pain by assessing these key somatic, cognitive, emotional, and behavioral responses that resemble the features of a traumatic stress reaction. Future prospective research with a sample of youth that are at-risk to develop chronic pain (e.g., youth undergoing major surgery) is needed to examine the predictive validity of the SPTS-P in the context of pediatric chronic pain.

Contrary to hypotheses, parent levels of SPT were not related to their child’s pain in our sample of youth who primarily presented with chronic primary pain (i.e., pain that is not associated with an underlying medical condition [27]). In addition to parent scores on the measure of PTSS being low, parent scores on the items of the SPTS-P were generally low, indicating that many parents in the current study did not endorse frequently responding to their child’s pain with somatic, cognitive, emotional, or behavioral responses similar to a traumatic stress reaction. SPT may present differently in parents whose children have other pain-related conditions, such as juvenile idiopathic arthritis or sickle cell disease. Indeed, parent SPT may be more related to the pain episodes (flare-ups or crises) that characterize these chronic conditions, particularly if the pain comes on suddenly, is severe, and lasts for an unpredictable duration. The current sample was also recruited from a tertiary pediatric chronic pain program, which suggests that many of these families likely have access to treatment providers specializing in pediatric pain as well as a variety of resources. As such, these parents may have lower levels of distress related to their child’s pain. Parent SPT may be more elevated among parents who do not feel supported or resourced in managing their child’s chronic pain. Parent SPT may also have a more distal relation to the youth’s pain experience, such that parent SPT influences the development and maintenance of youth chronic pain over time, perhaps through increased protective and monitoring behaviors. The theoretical and empirical literature on the social learning of pain suggests that these parenting behaviors may reinforce child pain behaviors and thus contribute to the onset and maintenance of chronic pain and disability in children [67,68,73,74,75,76]. In this way, parents with higher levels of SPT may engage in more maladaptive parenting behaviors when their child is in pain, and these behaviors may in turn impact the pain experience of youth. The cross-sectional nature of the current study precluded examination of this longitudinal relation. Further prospective research is needed to more comprehensively explore the relation between parent SPT and youth pain in a variety of clinical populations.

The current study had limitations that can be addressed in future research. First, the sample was typical of research samples recruited from tertiary pediatric chronic pain programs in North America in that the majority of youth and parents were White, female, and of high socioeconomic status. This limits the ability to generalize the results of the current study to other races or ethnicities, boys with chronic pain and their fathers, families with lower socioeconomic status, and families who are not receiving treatment in a tertiary pediatric chronic pain program. Second, only two parents reported their child’s chronic pain to be their worst traumatic event, and only 3.5% of parents met the clinical cut-off for PTSD. Both of these rates are lower than a previous study from the United States [2]. Future research is needed to validate the SPTS-P and evaluate parent SPT in diverse populations as the experience of parent SPT may vary across regions, races/ethnicities and socioeconomic backgrounds, mothers and fathers, or the specific nature of the child’s pain condition (e.g., headache vs. complex regional pain syndrome). Third, the current study only included one parent of the youth. As outlined by Palermo and Chambers [77], the influence of dyadic variables and family variables should be considered when individual parenting variables are examined in the context of pediatric chronic pain. For example, the level of SPT of the other parent may represent an unstudied third variable that may interact with the study parent’s SPT to moderate the relation with the youth’s pain experience. Future research should measure the SPT of both parents to examine the concordance of SPT levels in parents and their potential interactional effect on youth outcomes. Lastly, the current study presents a preliminary evaluation of the SPTS-P. Further research is needed to confirm its factor structure and psychometric properties.

## 5. Conclusions

The current study provides preliminary evidence of the reliability and validity of the parent version of the Sensitivity to Pain Traumatization Scale. Consistent with the original measure, the SPTS-P was found to have a one-factor structure, adequate to good reliability, and moderate convergent and discriminant validity in a sample of parents of youth with chronic pain. Parents with higher levels of SPT were more likely to respond to their child’s pain with protective and monitoring behaviors, highlighting the critical interaction between high levels of parent distress about child pain and maladaptive parenting responses to child pain. However, parent SPT was not related to their child’s pain experience. Further research is needed to validate the SPTS-P in other populations, including youth at-risk to develop chronic pain (e.g., youth undergoing major surgery) and youth from more diverse backgrounds.

## Figures and Tables

**Table 1:** Sociodemographic and pain characteristics of the sample, *N* = 170.

Characteristic	*M* (*SD*) Or *n* (%)
Parent age, *M* (*SD*) in years	45.03 (5.62)
Parent gender, *n* (%)	
Female	154 (90.6)
Male	15 (8.8)
Other	1 (0.6)
Parent race/ethnicity, *n* (%)	
White	145 (85.3)
Bi- or multi- racial	11 (6.5)
Arab/West Asian	4 (2.4)
Latin American	4 (2.4)
South Asian	2 (1.2)
Black/African American	1 (0.6)
Filipino	1 (0.6)
Other or do not want to answer	2 (1.2)
Parent marital status, *n* (%)	
Married	126 (74.1)
Separated or divorced	26 (15.3)
Common-law	8 (4.7)
Single	7 (4.1)
Widowed	3 (1.8)
Parent relationship to the child, *n* (%)	
Biological	166 (97.6)
Adoptive	2 (1.2)
Relative	1 (0.6)
Did not respond	1 (0.6)
Annual household income (CAD), *n* (%)	
≤$29,999	10 (5.9)
$30,000–$59,999	18 (10.6)
$60,000–$89,999	22 (12.9)
≥$90,000	98 (57.6)
Do not want to answer or did not respond	22 (12.9)
Parent education, *n* (%)	
High school or less	21 (12.4)
Vocational school or some college (no degree)	39 (22.9)
College or Bachelor’s degree	90 (52.9)
Graduate or professional degree	20 (11.8)
Youth age, *M* (*SD*) in years	14.32 (2.22)
Youth gender, *n* (%)	
Female	121 (71.2)
Male	46 (27.1)
Other	3 (1.8)
Youth race/ethnicity, *n* (%)	
White	136 (80.0)
Bi- or multi-racial	15 (8.8)
Arab/West Asian	4 (2.4)
South Asian	3 (1.8)
Black/African American	2 (1.2)
Latin American	2 (1.2)
Indigenous	1 (0.6)
Filipino	1 (0.6)
Other or do not want to answer	6 (3.5)
Youth total chronic pain duration, *M* (*SD*) in years	3.23 (3.08)
Youth pain frequency, *n* (%)	
Not at all	5 (2.9)
Once per week	13 (7.6)
2 to 3 times per week	48 (28.2)
4 to 6 times per week	23 (13.5)
Daily	81 (47.6)
Youth daily pain duration, *n* (%)	
Less than an hour	31 (18.2)
A few hours	40 (23.5)
Half the day	28 (16.5)
All day	68 (40.0)
Did not answer	3 (1.8)
Youth pain locations, *n* (%)	
Head	124 (72.9)
Muscle and joints	43 (25.3)
Stomach	33 (19.4)
Legs	24 (14.1)
Chest	21 (12.4)
Other	41 (24.1)

Note. Youth were able to select more than one pain location.

**Table 2:** Types of traumatic events reported by parents.

Traumatic Event	*n* (%)
Death	49 (29.2)
Physical illness, hospitalization or medical emergency	26 (15.5)
Accident	21 (12.5)
Sexual abuse	15 (8.9)
Physical abuse	10 (6.0)
Divorce	10 (6.0)
Other	8 (4.8)
Natural disaster	6 (3.6)
Family-related conflict	5 (3.0)
Mental illness	4 (2.4)
Verbal conflict or abuse	4 (2.4)
Fire	3 (1.8)
Child’s chronic pain	2 (1.2)
Suicide attempt	2 (1.2)
Substance abuse	2 (1.2)
Social difficulties	1 (0.6)

Note. Parents were asked to report their worst event. However, parents could record more than one event or more than one type of traumatic event. Thus, percentages are based on the sum of types of traumatic events reported by parents (*N* = 170). Fourteen parents (8.2%) did not report an event (i.e., left the textbox blank). Among parents who reported an event, two (1.3%) did not identify a traumatic event (e.g., wrote “not applicable”), 142 (91.0%) identified one event or one type of event, and 12 (7.7%) identified more than one event or type of event.

**Table 3:** Item-total statistics and factor loadings for the one-factor solution of the SPTS-P.

SPTS-P Item	*M* (*SD*)	*α*-if-Item-Deleted	Inter-Total *r*	Factor Loading	Communality
1. When my child is in pain, it keeps me awake at night.	1.34 (1.15)	0.841	0.631	0.663	0.440
2. When my child is in pain, everything I see or do reminds me of their pain.	0.48 (0.86)	0.843	0.635	0.778	0.606
3. I try to avoid having my child do activities that cause him/her pain.	1.41 (1.26)	0.857	0.453	0.491	0.241
4. When my child is in pain, I’m scared that it’s the beginning of a terrible problem.	0.74 (0.94)	0.844	0.608	0.700	0.489
5. My child’s pain seems to bother me more than other parents are bothered by their children’s pain.	0.45 (0.88)	0.845	0.594	0.740	0.548
6. When my child is in pain, I think about it even when I don’t mean to.	0.82 (1.01)	0.838	0.688	0.770	0.592
7. I can’t stand when my child is in pain.	2.00 (1.38)	0.852	0.529	0.508	0.258
8. When my child is in pain, I feel distant from people even when I’m talking to them.	0.51 (0.87)	0.847	0.576	0.710	0.504
9. As soon as my child’s pain comes on, I give him/her medications to reduce it.	1.34 (1.26)	0.868	0.307	0.355	0.126
10. My child’s pain terrifies me.	0.61 (0.98)	0.844	0.600	0.748	0.560
11. When my child is in pain, things don’t feel real.	0.22 (0.63)	0.852	0.508	0.688	0.473
12. I feel sick to my stomach when my child is in pain.	0.37 (0.69)	0.848	0.583	0.744	0.553

**Table 4:** Means (*M*), standard deviations (*SD*), and Spearman correlations among the SPTS-P and related constructs.

Variable	1	2	3	4	5	6	*M* (*SD*)
1. SPTS-P	-						10.29 (7.64)
2. ASI-3	0.298 ***	-					8.96 (9.53)
3. PASS-20	0.393 ***	0.537 ***	-				18.82 (14.67)
4. PCS-P	0.742 ***	0.295 ***	0.311 ***	-			13.78 (8.70)
5. PCL-5	0.310 ***	0.386 ***	0.402 ***	0.283 ***	-		9.51 (10.95)
6. HADS-D	0.199 **	0.467 ***	0.402 ***	0.206 **	0.620 ***	-	3.29 (3.34)
7. HADS-A	0.268 ***	0.531 ***	0.396 ***	0.210 **	0.512 ***	0.623 ***	5.97 (3.71)

Note. *** *p* < 0.001, ** *p* < 0.01. Abbreviations: SPTS-P, Sensitivity to Pain Traumatization Scale-Parent Version; ASI-3, Anxiety Sensitivity Index-3; PASS-20, Pain Anxiety Symptoms Scale-Short Version; PCS-P, Pain Catastrophizing Scale-Parent Version; PCL-5, PTSD Checklist for DSM-5; HADS-D, Hospital Anxiety and Depression Scale-Depression subscale; HADS-A, Hospital Anxiety and Depression Scale-Anxiety subscale.

**Table 5:** Means (*M*), standard deviations (*SD*), and Spearman correlations among the SPTS-P and measures for construct validity.

Variable	SPTS-P	2	3	4	5	*M* (*SD*)
2. ARCS-P	0.608 ***	-				1.38 (0.73)
3. ARCS-M	0.458 ***	0.504 ***	-			2.87 (0.84)
4. Youth Pain Intensity	−0.024	−0.030	−0.121	-		5.49 (1.85)
5. Youth Pain Unpleasantness	0.055	0.026	−0.047	0.615 ***	-	2.01 (0.90)
6. Youth Pain Interference	0.068	0.024	−0.101	0.502 ***	0.552 ***	55.48 (9.12)

Note. *** *p* < 0.001. Abbreviations: SPTS-P, Sensitivity to Pain Traumatization Scale-Parent Version; ARCS-P, Adult Responses to Children’s Symptoms-Protect subscale; ARCS-M, Adult Responses to Children’s Symptoms-Monitor subscale.

## Data Availability

Data available upon request from corresponding author.
